# Building a unified trauma data repository: bridging military and civilian research to transform care

**DOI:** 10.1136/tsaco-2025-002086

**Published:** 2026-07-02

**Authors:** James Boyce Phillips, Pamela J Bixby, Michelle A Price

**Affiliations:** 1 Defense Health Agency, US Department of Defense, Fort Detrick, Maryland, USA; 2 Coalition for National Trauma Research, San Antonio, Texas, USA

**Keywords:** Efficiency, Research, Surveys And Questionnaires

 Following the success of the Federal Interagency Traumatic Brain Injury Research (FITBIR) Information System in sharing data across the entire traumatic brain injury research field and facilitating collaboration between laboratories, the Defense Health Agency’s Research and Engineering Combat Casualty Care Portfolio recognized that a data repository for general trauma research could increase the relevance and value of the data collected, benefiting trauma care, leading to significant advances in treatment, and improving outcomes in both military and civilian populations.

With the support of Congressional Community Project Funding, the US Department of Defense awarded a contract to the Coalition for National Trauma Research (CNTR) to integrate its National Trauma Research Repository (NTRR) with FITBIR. This integration enables cross-platform data aggregation with great promise for unique analyses that can close gaps in trauma care knowledge. CNTR engaged a multidisciplinary Steering Committee (SC) including both military and civilian trauma surgeons, multi-institutional trials experts, and data scientists to identify common data elements (CDEs) found in general trauma investigations and align them with existing elements already specified in FITBIR to the greatest extent possible.

**Figure 1:**
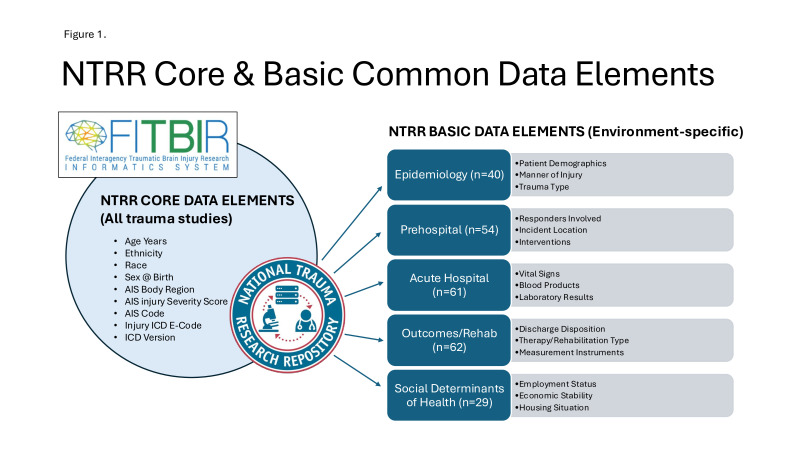
National Trauma Research Repository (NTRR) core and basic common data elements. AIS, Abbreviated Injury Scale; ICD, International Classification of Diseases.

The resulting NTRR data dictionary includes 10 core CDEs along with their definitions and permissible values, which we recommend all trauma studies collect for the broadest harmonization possible. This brief list of foundational elements includes: age (years), ethnicity, race, sex at birth, Abbreviated Injury Scale (AIS) body region, AIS dictionary version type, AIS Injury Severity Score, AIS code, injury International Classification of Diseases (ICD) E-code and ICD version. Additionally, CNTR convened five workgroups of subject matter experts to evaluate sets of basic CDEs specific to the study environments of epidemiology, prehospital, acute hospital, rehabilitation/outcomes, and social determinants of health (figure 1). While neither the core nor any of the basic elements are required to be collected, the SC strongly recommends that investigators adopt the core to ensure maximum utility of their data and review and adopt those basic elements that align with their study context. The consistent use of CDEs facilitates data sharing, reduces duplication of effort, and enhances the ability to conduct large-scale analyses and meta-analyses.

We encourage you to read the manuscripts in this special issue^1^ ^2^ ^3^ ^4^ ^5^ ^6^ outlining the work of these bodies to develop the growing data dictionary now available in the NTRR (NTRR.NIH.GOV), and to make the broadest possible use of this new resource designed to increase our knowledge, change practice, and improve outcomes in both military and civilian arenas.
